# Correlational data concerning body centre of mass acceleration, muscle activity, and forces exerted during a suspended lunge under different stability conditions in high-standard track and field athletes

**DOI:** 10.1016/j.dib.2019.104912

**Published:** 2019-11-30

**Authors:** Joan Aguilera-Castells, Bernat Buscà, Jordi Arboix-Alió, Gary McEwan, Julio Calleja-González, Javier Peña

**Affiliations:** a Faculty of Psychology, Education Sciences and Sport Blanquerna, Ramon Llull University, Barcelona, Spain; b School of Health and Life Sciences, University of the West of Scotland, Glasgow, United Kingdom; c Department of Physical Activity and Sport Science, University of Basque Country, Alava, Spain; d Sport and Physical Activity Studies Centre (CEEAF), University of Vic – Central University of Catalonia, Barcelona, Spain

**Keywords:** Suspension training, Lower limb, Instability, Electromyography, Strength

## Abstract

This article reports data concerning the body centre of mass acceleration, muscle activity, and forces exerted during a suspended lunge under different stability conditions. Ten high-standard track and field athletes were recruited to perform one set of 5 repetitions of the following exercises: suspended lunge, suspended lunge-Foam (front leg on a foam balance-pad and the rear leg on the suspension cradles), a suspended lunge-BOSU up (dome side up), and a suspended lunge-BOSU down (dome side down). For each exercise trial, the acceleration of the body centre of mass (tri-axial accelerometer BIOPAC), the muscle activity of the front leg (surface electromyography BIOPAC) and the force exerted on the suspension strap (load cell Phidgets) were measured. The data revealed that the intra-reliability of the data range from good (ICC: 0.821) to excellent (ICC: 0.970) in all dependent variables and exercise conditions. Besides, the Pearson correlation between muscle activity and the body centre of mass acceleration showed a significant positive correlation for all the exercises and analysed muscles (range from *r* = 0.393 to *r* = 0.826; *p* < 0.05) with moderate to very large effect, except for the rectus and biceps femoris. Moreover, the force exerted on the suspension strap significantly correlated with the body centre of mass acceleration in all the exercises (range from *r* = −0.595 to *r* = −0.797, *p* < 0.05) with a very large effect, except for the suspension lunge that registered a large effect.

**Table:** Specifications Table

Subject	Sport sciences
Specific subject area	Strength and conditioning
Type of data	Table Image Figure
How data were acquired	Six channels of sEMG (Biopac), tri-axial accelerometer (Biopac) and s-type load cell (Phidgets) acquired using Biopac System MP-150 at a sampling rate of 1.0 kHz.
Data format	Raw Filtered Analysed
Parameters for data collection	Participants (high-standard athletes) were excluded if they presented any injuries or pain related to cardiovascular, musculoskeletal, or neurological disorders. All subjects were instructed to refrain from high-intensity physical activity or neuromuscular stimulation for the 24h before the experimental sessions, and they consumed no food, drinks, or stimulants (i.e., caffeine) 4h before testing.
Description of data collection	The experiment was conducted in 2 sessions: familiarisation and experimental. They were performed at the same time in the morning, separated by a week. All suspended lunge conditions were executed using a TRX Suspension Trainer™ device. An S-Type Load Cell was used to measure the force exerted on the suspension strap by the suspended lower limb in random order (90-s rest). The load cell was displayed on the suspension device. Surface electromyography (sEMG) was used to measure muscle activity in the dominant leg (6 most recruited muscles), which was established as the front leg. The tri-axial accelerometer was placed in the waist to measure the body centre of mass acceleration.
Data source location	Barcelona (Catalonia) Spain
Data accessibility	Repository name: Mendeley Data Direct URL to data: https://doi.org/10.17632/8wj8gpgwmr.3

**Table:** 

**Value of the Data** • The presented data might improve the understanding of the acceleration contribution to muscle involvement, and the forces exerted in a lower limb suspended exercise commonly used in specific strength and conditioning programs. • Strength and conditioning coaches and practitioners could use the data to select different variations of a suspended unilateral lower limb exercise. • The different correlations associating muscle activity and forces exerted in different exercise conditions could be used to analyse the ability of a subject to stabilizing a unilateral lower-limb action. • Additionally, data might help sports facilities to select the best equipment for creating unstable strength and conditioning environments.

## 1 Data

The present article contains data concerning body centre of mass acceleration, muscle activity and forces exerted during the execution of a suspended lunge exercise under different conditions of instability in high-standard athletes (athletes enrolled in a sports talent program, national finalists and training 10 hours weekly, see Table 1). Different variables were measured by using surface electromyography (sEMG), a Tri-axial accelerometer and a load cell simultaneously recorded by the BIOPAC MP-150 at a sampling rate of 1.0 kHz (BIOPAC System, INC., Goleta, CA). Reliability of the data is reported in Table 2. The correlation between the sEMG signals for all analysed muscles and acceleration are reported in Table 3. Correlations among the forces exerted on the suspended strap and acceleration are reported in Table 4. The smallest worthwhile change (SWC) and the coefficient of variation of the dependent variables for each condition are reported in Table 5. Regression point plots expressing the relationship between the acceleration and muscle activity of the rectus femoris, vastus medialis, vastus lateralis, gluteus maximus, gluteus medius and biceps femoris are shown in Fig. 1, Fig. 2, Fig. 3, Fig. 4, Fig. 5 and Fig. 6, respectively. Fig. 7 shows the regression point plots between the acceleration and force exerted on the suspension strap.

**Table 1:** Participants' characteristics including athletic background.

Participant	Age	Height (m)	Weight (kg)	Training age	Athletic level	Athletic discipline	Hours of training		
							Weekly	Training specifications	
Sub1	22	1.69	57	16	Int.	Endurance (800 m)	10	S: 3 Sp: 3	E: 3 T: 1
Sub2	19	1.79	71	13	Int.	Endurance (800 m)	10	S: 3 Sp: 3	E: 3 T: 1
Sub3	21	1.76	63	15	Int.	Sprint (400 m)	10	S: 3 Sp: 3	E: 3 T: 1
Sub4	21	1.70	64	15	Int.	Sprint (400 m)	10	S: 3 Sp: 3	E: 3 T: 1
Sub5	18	1.70	58	12	Int.	Sprint (400 m)	10	S: 3 Sp: 3	E: 3 T: 1
Sub6	20	1.71	63	15	Int.	Sprint (400 m)	10	S: 3 Sp: 3	E: 3 T: 1
Sub7	18	1.68	60.5	13	Int.	Sprint (100 m)	10	S: 3 Sp: 3	E: 3 T: 1
Sub8	18	1.65	49	12	Int.	Sprint (400 m)	10	S: 3 Sp: 3	E: 3 T: 1
Sub9	21	1.67	51	15	Int.	Endurance (800 m)	10	S: 3	E: 3 T: 1
Sub10	20	1.67	55	15	Int.	Sprint (400 m)	10	S: 3	E: 3 T: 1
**Mean**	19.80	1.70	59.15	14.10					
**SD**	1.48	0.04	6.57	1.45					

Sub: Subject; Int: International; S: Strength; Sp: Speed; E: Endurance; T: Technique.

**Table 2:** Reliability values for each muscle analysed, acceleration and force under suspended lunge conditions.

	Exercise Condition	ICCs (level of reliability)	95% CI	SEM
Rectus femoris	SL	0.876 (Good)	0.65–0.97	0.06
	SL_Foam	0.873 (Good)	0.62–0.97	0.06
	SL_BU	0.844 (Good)	0.67–0.97	0.07
	SL_BD	0.963 (Excellent)	0.89–0.99	0.04
Vastus medialis	SL	0.879 (Good)	0.64–0.97	0.04
	SL_Foam	0.923 (Excellent)	0.78–0.98	0.04
	SL_BU	0.920 (Excellent)	0.77–0.98	0.05
	SL_BD	0.844 (Good)	0.56–0.96	0.06
Vastus lateralis	SL	0.821 (Good)	0.46–0.95	0.05
	SL_Foam	0.888 (Good)	0.68–0.97	0.04
	SL_BU	0.903 (Excellent)	0.73–0.97	0.05
	SL_BD	0.857 (Good)	0.57–0.96	0.05
Gluteus maximus	SL	0.940 (Excellent)	0.83–0.98	0.04
	SL_Foam	0.945 (Excellent)	0.83–0.99	0.03
	SL_BU	0.960 (Excellent)	0.89–0.99	0.05
	SL_BD	0.939 (Excellent)	0.83–0.98	0.06
Gluteus medius	SL	0.846 (Good)	0.53–0.96	0.07
	SL_Foam	0.912 (Excellent)	0.75–0.98	0.06
	SL_BU	0.916 (Excellent)	0.76–0.98	0.09
	SL_BD	0.896 (Good)	0.69–0.97	0.09
Biceps femoris	SL	0.844 (Good)	0.54–0.96	0.04
	SL_Foam	0.964 (Excellent)	0.90–0.99	0.01
	SL_BU	0.936 (Excellent)	0.82–0.98	0.03
	SL_BD	0.905 (Excellent)	0.72–0.97	0.04
Acceleration	SL	0.990 (Excellent)	0.96–1	0.01
	SL_Foam	0.994 (Excellent)	0.98–1	0.01
	SL_BU	0.996 (Excellent)	0.99–1	0.01
	SL_BD	0.996 (Excellent)	0.99–1	0.01
Force	SL	0.964 (Excellent)	0.90–0.99	1.06
	SL_Foam	0.969 (Excellent)	0.91–0.99	1.02
	SL_BU	0.961 (Excellent)	0.89–0.99	1.16
	SL_BD	0.970 (Excellent)	0.92–0.99	1.08

CI: Confidence interval; ICCs: Interclass correlation coefficients; SEM: Standard error of measurement; SL: Suspended lunge; SL_Foam: Suspended lunge-Foam; SL_BU: Suspended lunge-BOSU up; SL_BD: Suspended lunge-BOSU down.

**Table 3:** Pearson's correlation between muscle activity values for each muscle analysed and acceleration under suspended lunge conditions.

	Suspended lunge	Suspended lunge-Foam	Suspended lunge-BOSU up	Suspended lunge-BOSU down
Rectus femoris	−0.050	0.192	0.283	−0.087
*p*-value	0.794	0.310	0.130	0.649
LC	Trivial	Small	Small	Trivial
Vastus medialis	0.699*	0.632*	0.650*	0.588*
*p*-value	0.000	0.000	0.000	0.001
LC	Large	Large	Large	Large
Vastus lateralis	0.393*	0.689*	0.629*	0.506*
*p*-value	0.031	0.000	0.000	0.004
LC	Moderate	Large	Large	Large
Gluteus maximus	0.477*	0.553*	0.611*	0.558*
*p*-value	0.008	0.002	0.000	0.001
LC	Moderate	Large	Large	Large
Gluteus medius	0.526*	0.749*	0.826*	0.646*
*p*-value	0.003	0.000	0.000	0.000
LC	Large	Very large	Very large	Large
Biceps femoris	0.468*	−0.216	0.250	−0.158
*p*-value	0.009	0.251	0.183	0.403
LC	Moderate	Small	Small	Small

LC: Level of correlation; *Statistical significance at *p* < 0.05.

**Table 4:** Pearson's correlation (*r*) between forces exerted on the suspension strap and acceleration under suspended lunge conditions.

	Suspended lunge	Suspended lunge-Foam	Suspended lunge-BOSU up	Suspended lunge-BOSU down
*r*	−0.595*	−0.797*	−0.776*	−0.741*
*p*-value	0.001	0.000	0.000	0.000
LC	Large	Very large	Very large	Very large

LC: Level of correlation; *Statistical significance at *p* < 0.05.

**Table 5:** Smallest worthwhile change and coefficient of variation values for each muscle analysed, acceleration and force under suspended lunge conditions.

	Exercise Condition	SWC	CV
Rectus femoris	SL	0.03	0.002
	SL_Foam	0.03	0.002
	SL_BU	0.04	0.002
	SL_BD	0.04	0.002
Vastus medialis	SL	0.02	0.001
	SL_Foam	0.03	0.001
	SL_BU	0.03	0.002
	SL_BD	0.03	0.001
Vastus lateralis	SL	0.02	0.001
	SL_Foam	0.02	0.001
	SL_BU	0.03	0.002
	SL_BD	0.03	0.001
Gluteus maximus	SL	0.04	0.002
	SL_Foam	0.03	0.001
	SL_BU	0.05	0.003
	SL_BD	0.05	0.002
Gluteus medius	SL	0.03	0.002
	SL_Foam	0.04	0.002
	SL_BU	0.06	0.003
	SL_BD	0.06	0.003
Biceps femoris	SL	0.02	0.001
	SL_Foam	0.01	0.001
	SL_BU	0.02	0.001
	SL_BD	0.03	0.001
Acceleration	SL	0.02	0.001
	SL_Foam	0.02	0.001
	SL_BU	0.03	0.001
	SL_BD	0.03	0.001
Force	SL	1.11	0.056
	SL_Foam	1.15	0.058
	SL_BU	1.18	0.059
	SL_BD	1.25	0.062

SWC: Smallest worthwhile change; CV: Coefficient of variation; SL: Suspended lunge; SL_Foam: Suspended lunge-Foam; SL_BU: Suspended lunge-Bosu up; SL_BD: Suspended lunge-Bosu down.

**Fig. 1:**
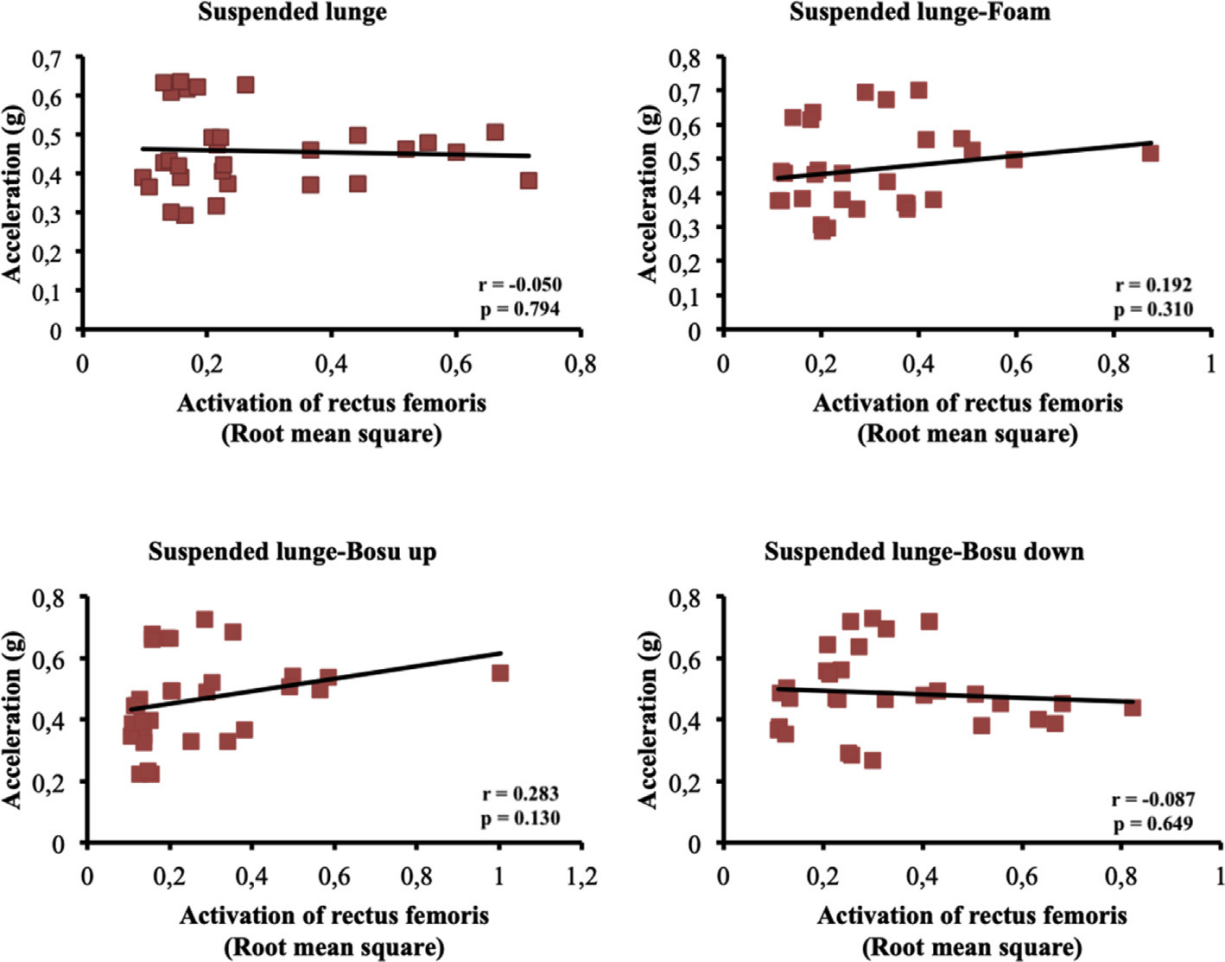
Correlation between rectus femoris activation and acceleration values under suspended lunge conditions.

**Fig. 2:**
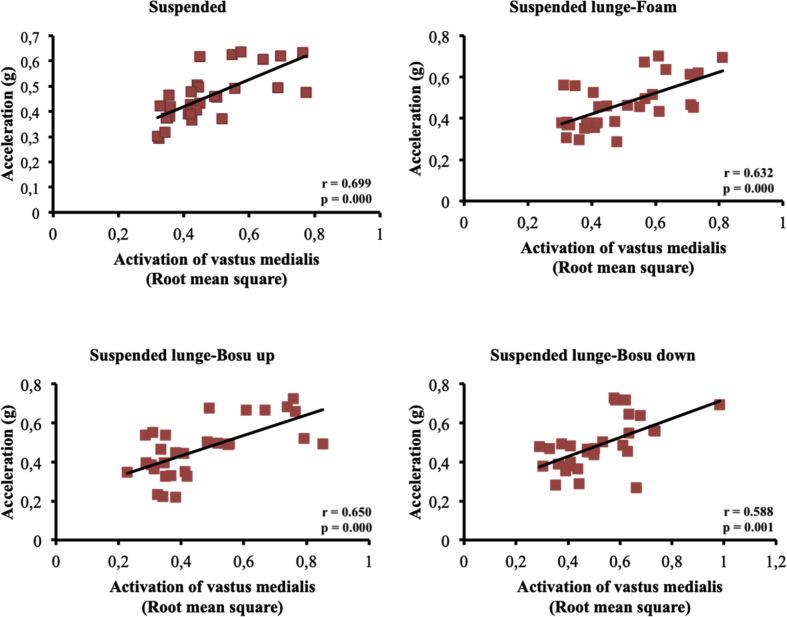
Correlation between vastus medialis activation and acceleration values under suspended lunge conditions.

**Fig. 3:**
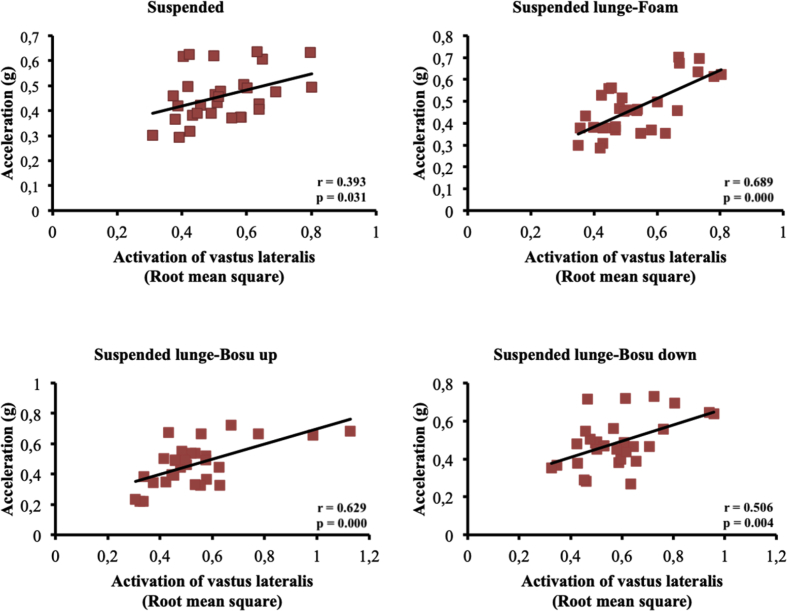
Correlation between vastus lateralis activation and acceleration values under suspended lunge conditions.

**Fig. 4:**
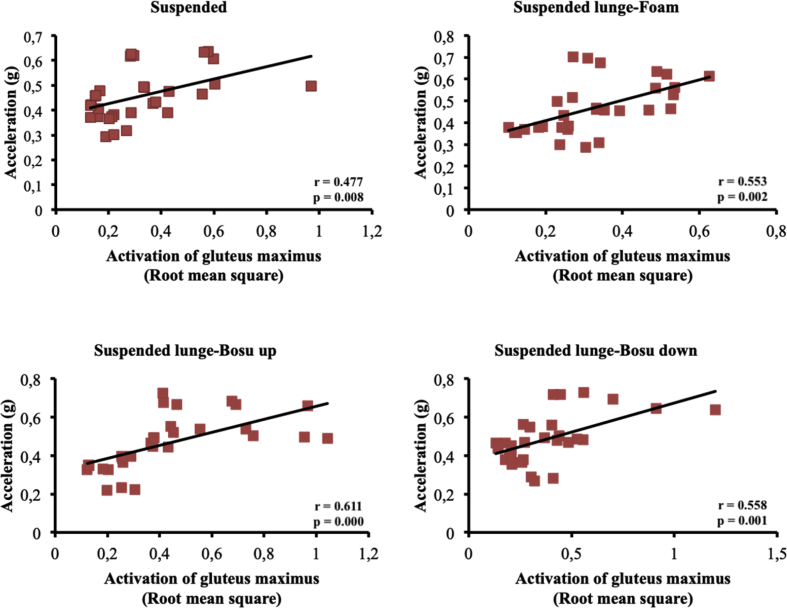
Correlation between gluteus maximus activation and acceleration values under suspended lunge conditions.

**Fig. 5:**
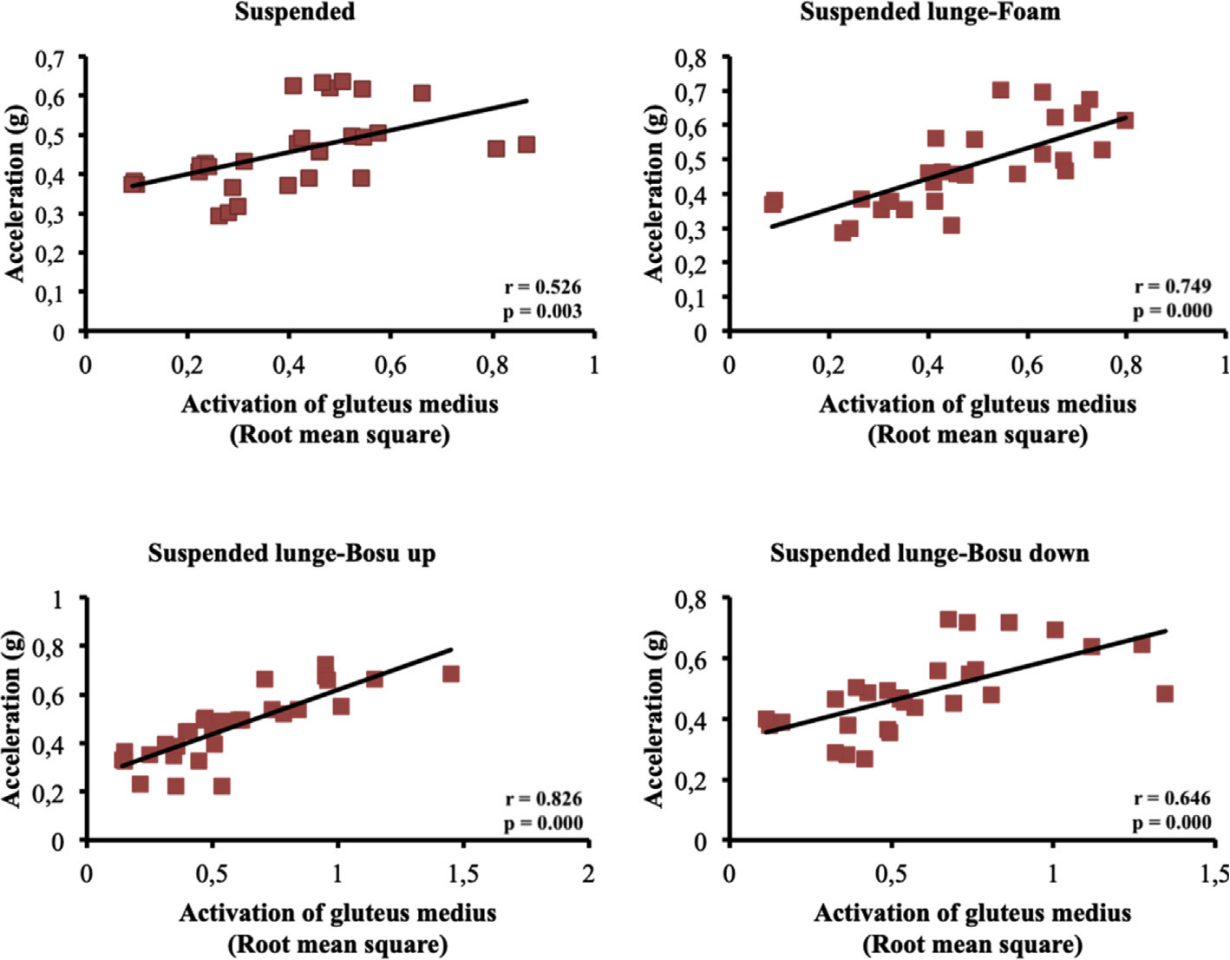
Correlation between gluteus medius activation and acceleration values under suspended lunge conditions.

**Fig. 6:**
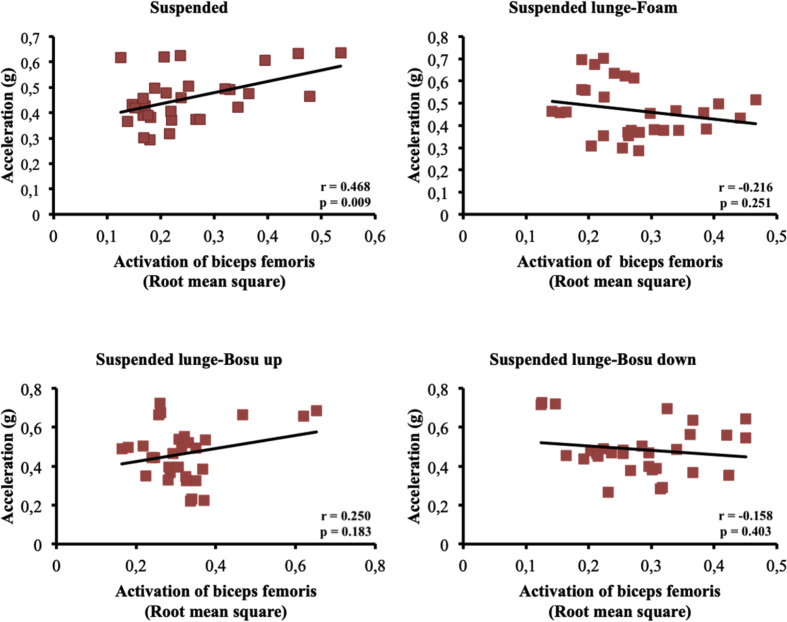
Correlation between biceps femoris activation and acceleration values under suspended lunge conditions.

**Fig. 7:**
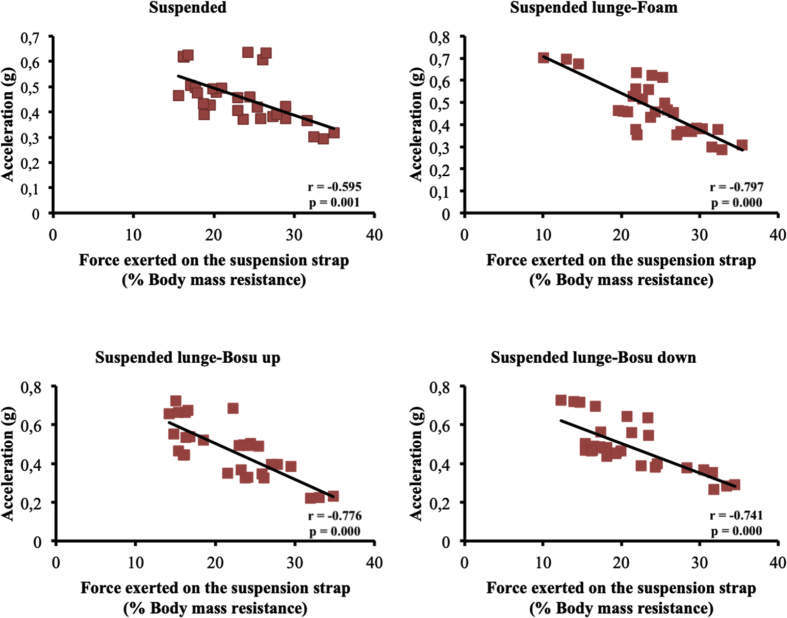
Correlation between forces exerted on the suspension strap and acceleration values under suspended lunge conditions.

## 2 Experimental design, materials, and methods

A repeated measures design was used to establish the relationship between the body centre of mass acceleration, muscle activity and the force exerted on the suspension strap during different suspended lunge conditions. Ten high-standard track and field athletes (mean ± standard deviation (SD): age, 19.8 ± 1.48 years; height, 1.70 ± 0.04 m; body mass, 59.15 ± 6.67 Kg) were recruited to perform a suspended lunge in 4 conditions: a) suspended lunge (front leg on the floor and the rear leg leaning within the suspension device cradle (TRX® Suspension training system, patent No.: US 7,044,896 B2; Fitness Anywhere, San Francisco, CA), b) suspended foam (same as the previous exercise with the front leg on a balance-pad (AIREX®, Sins, CH), c) suspended BOSU up (front leg on the BOSU (BOSU®, Ashland, OH) with the dome side up), and d) suspended BOSU down (same as the previous exercise) with the dome side down. Participants assumed a lunge position with their arms crossed on their chest, and their upper body upright with a lower back natural sway. For the lower body, the subjects lowered the body (eccentric phases) until the forward knee flexed to 90^°^ and then, returned to the starting position with a full knee extension of the forward leg (concentric phase) [1]. The vertical displacement during all exercises was measured with a positional encoder (WSB 16K-200; ASM Inc., Moosinning, DE) and the tether of the positional encoder was attached to the hip. The forward food was placed on different surfaces (floor, balance pad, BOSU dome side up and down) with the heel contact on the floor, balance pad or BOSU. The forward leg was chosen as the dominant leg, which was determined by asking participants which leg they would use to kick a ball [2]. The rear foot was placed within the suspension device cradle with slight plantar flexion in all the exercise conditions (supplementary material). Besides, the height and stepped distance, and 90^°^ of knee flexion were normalized. The height of the suspension straps was established as 60% of the subject's leg length, and the subjects stepped distance was normalized to 80% of their leg length [3]. The 90^°^ of knee flexion were established by measuring with a manual goniometer the knee flexion in the lower position. Once the 90^°^ were identified, customized stoppers (similar to hurdles) were used to fix this position. Feedback on how much they had to go down, and when to start the countermovement was also provided to the participants (see Supplemental material). Before the exercise trials, a standardized warm-up was carried out, consisting of 5 minutes of cycling with 100 W of cadence maintaining 60 revolutions per minute. Then, each participant performed a set of 5 consecutive repetitions of each suspended lunge exercise. The objective was to perform the different tasks at a controlled pace, maintaining the posture as consistently as possible. During the exercise trials, all subjects performed one set of 5 repetitions of each condition with a standardized pace of 70 beats per minute in a randomized order. Participants were provided with a 90-s rest between exercises to avoid fatigue.

During the trials muscle activity, forces exerted on the suspension strap and body centre of mass acceleration were measured. To record muscle activity, 12 bipolar surface electromyography electrodes were placed on the front leg (dominant leg) on the rectus femoris, vastus lateralis, vastus medialis, gluteus maximus, gluteus medius and biceps femoris following the SENIAM Project recommendations [4]. An additional electrode was placed directly over the right anterior iliac spine as a ground surface electrode. The surface electromyographic values (root mean square) were registered with a BIOPAC MP-150 at a sampling rate of 1.0 kHz. The signal was bandpass filtered at 50–500 Hz while utilizing a 4th Butterworth filter and then analysed using the AcqKnowledge 4.2 software (BIOPAC System, INC., Goleta, CA). The forces exerted on the suspension strap were recorded using an S-Type Load Cell (model CZL301C; Phidgets Inc., Alberta, CAN) with a sample rate of 200 Hz. The load cell was placed between the anchor point (2.95 m from the ground) and the suspension straps. Moreover, a tri-axial accelerometer (model TSD109F, BIOPAC System, INC., Goleta, CA) was placed in the waist to measure the body centre of mass accelerations with a sample rate of 2.0 kHz, a sensitivity of 40 mV/g, and a range of ±50g. The force and body centre of mass acceleration were recorded using a BIOPAC MP-150 and its original software.

Surface electromyography, force and body centre of mass acceleration signals for each exercise condition were analysed by taking the average of the three middle repetitions, excluding the first and fifth repetitions from data analysis. To normalize the force exerted on the suspension straps, an equation was used for each participant based on load and body mass (%_body mass resistance = load/bodyweight x 100) [5]. The number of participants recruited was established using an α level of 0.05 and setting power at 0.50 using G Power Software (University of Dusseldorf). The Shapiro-Wilk test was carried out to confirm that data were normally distributed to approve the use of parametric techniques. The intra-rater reliability of all the dependent variables was assessed using an intraclass correlation coefficient (ICC), and their 95% confidence interval based on mean-rating (K = 3), absolute-agreement, two-way mixed-effects model. Pearson's correlation (r) was employed to determine the relationship between the following dependent variables a) muscle activity and body centre of mass acceleration, and b) force exerted on the suspension straps and body centre of mass acceleration. The ICC was interpreted such as poor (<0.5), moderate (0.5–0.75), good (0.75–0.90), or excellent (>0.90) reliability [6]. The coefficient of variation was also estimated, and the small-standardized effect based on Cohen's effect size principle (SWC) was calculated as 0.2 x between-subject standard deviation (SD).

Additionally, the magnitude of the Pearson's correlation values were interpreted as <0.2 = trivial; 0.2–0.6 = small; 0.6–1.2 = moderate; 1.2–2.0 = large; >2.0 = very large [7]. Significance was accepted when *p* value was <0.05. The statistical analysis was accomplished using SPSS (Version 20 for Mac; SPSS Inc., Chicago, IL, USA).

## Figures and Tables

**Figs1:**
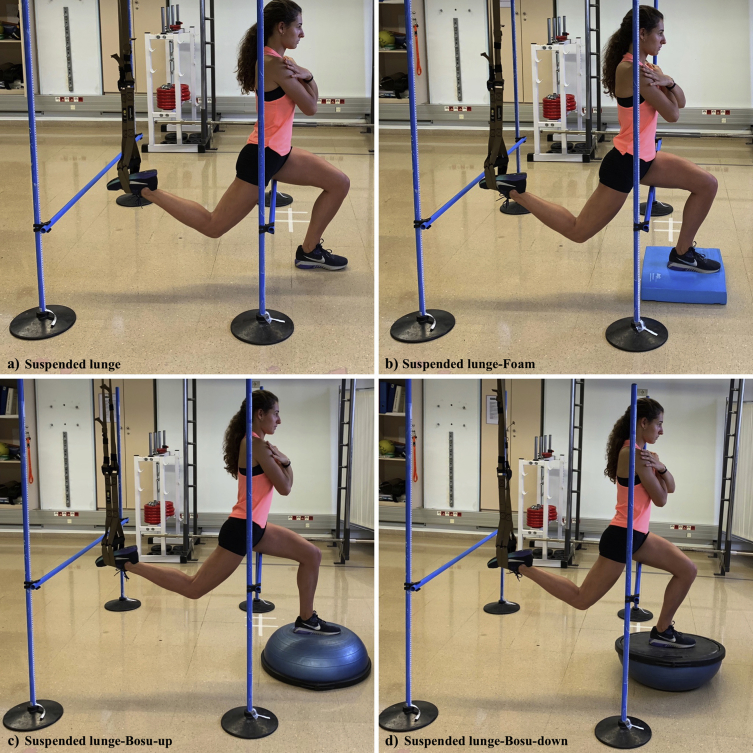

