# Supplementary material for: Correlational data concerning body centre of mass acceleration, muscle activity, and forces exerted during a suspended lunge under different stability conditions in high-standard track and field athletes
Source: Data Brief. 2019 Nov 30;28:104912. doi: 10.1016/j.dib.2019.104912 (PMC6920397; doi:10.1016/j.dib.2019.104912)
Supplement: Multimedia component 1 [file mmc1.pdf]

Author declaration

[Instructions: Please check all applicable boxes and provide additional information as requested.]

1. Conflict of Interest

Potential conflict of interest exists:

We wish to draw the attention of the Editor to the following facts, which may be considered as potential conflicts of interest, and to significant financial contributions to this work:

The nature of potential conflict of interest is described below:

☒No conflict of interest exists.

We wish to confirm that there are no known conflicts of interest associated with this publication and there has been no significant financial support for this work that could have influenced its outcome.

2. Funding

☒Funding was received for this work.

All of the sources of funding for the work described in this publication are acknowledged below:

The research was supported by the Secretariat of University and Research of the Ministry of Business and Knowledge of the Government of Catalonia and the European Social fund under Grant [2019 FI_B1 00165] and l'Obra Social 'la Caixa'. The funders had no role in study design, data collection and analysis, decision to publish or preparation of the manuscript.

☐No funding was received for this work.

3. Intellectual Property

☒We confirm that we have given due consideration to the protection of intellectual property associated with this work and that there are no impediments to publication, including the timing of publication, with respect to intellectual property. In so doing we confirm that we have followed the regulations of our institutions concerning intellectual property.

4. Research Ethics

☒We further confirm that any aspect of the work covered in this manuscript that has involved human patients has been conducted with the ethical approval of all relevant bodies and that such approvals are acknowledged within the manuscript.

☒IRB approval was obtained (required for studies and series of 3 or more cases)

☒Written consent to publish potentially identifying information, such as details or the case and photographs, was obtained from the patient(s) or their legal guardian(s).

5. Authorship

The International Committee of Medical Journal Editors (ICMJE) recommends that authorship be based on the following four criteria:

1. Substantial contributions to the conception or design of the work; or the acquisition, analysis, or interpretation of data for the work; AND
2. Drafting the work or revising it critically for important intellectual content; AND
3. Final approval of the version to be published; AND
4. Agreement to be accountable for all aspects of the work in ensuring that questions related to the accuracy or integrity of any part of the work are appropriately investigated and resolved.

All those designated as authors should meet all four criteria for authorship, and all who meet the four criteria should be identified as authors. For more information on authorship, please see http://www.icmje.org/recommendations/browse/roles-and-responsibilities/defining-the-role-of-authors-and-contributors.html#two.

☒All listed authors meet the ICMJE criteria. We attest that all authors contributed significantly to the creation of this manuscript, each having fulfilled criteria as established by the ICMJE.

☐One or more listed authors do(es) not meet the ICMJE criteria.

We believe these individuals should be listed as authors because:

They committed with the four criteria recommended by the ICMJE. The table (below) shows their substantial contribution in the different phases of the research.

	Concept and study design	Data acquisition	Data analysis and interpretation	Reviewing/ editing a draft of the manuscript	Final approval of the manuscript
**Joan Aguilera-Castells**	X	X	X	X	X
**Bernat Buscà**	X	X	X	X	X
**Jordi Arboix-Alió**	X	X	X	X	X
**Gary McEwan**	X	X	X	X	X

**Julio Calleja-González**	x	x	x	x	x
**Javier Peña**	x	x	x	x	x

☒We confirm that the manuscript has been read and approved by all named authors.

☒We confirm that the order of authors listed in the manuscript has been approved by all named authors.

6. Contact with the Editorial Office

The Corresponding Author declared on the title page of the manuscript is:

Dr. Bernat Buscà

☒This author submitted this manuscript using his/her account in EVISE.

☒We understand that this Corresponding Author is the sole contact for the Editorial process (including EVISE and direct communications with the office). He/she is responsible for communicating with the other authors about progress, submissions of revisions and final approval of proofs.

☒We confirm that the email address shown below is accessible by the Corresponding Author, is the address to which Corresponding Author's EVISE account is linked, and has been configured to accept email from the editorial office of American Journal of Ophthalmology Case Reports:

bernatbs@blanquerna.url.edu

☐Someone other than the Corresponding Author declared above submitted this manuscript from his/her account in EVISE:

[Insert name below]

☐We understand that this author is the sole contact for the Editorial process (including EVISE and direct communications with the office). He/she is responsible for communicating with the other authors, including the Corresponding Author, about progress, submissions of revisions and final approval of proofs.

We the undersigned agree with all of the above.

Author’s name (Fist, Last)	Signature	Date
1. Joan Aguilera-Castells	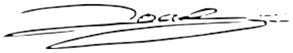	29/10/2019
2. Bernat Buscà	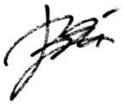	29/10/2019
3. Jordi Arboix-Alió	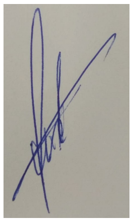	29/10/2019
4. Gary McEwan	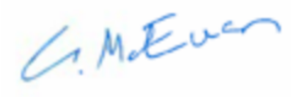	29/10/2019
5. Julio Calleja-González	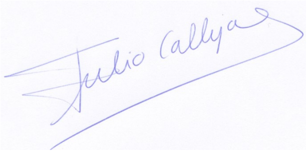	29/10/2019
6. Javier Peña	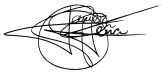	29/10/2019
